# Novel Evidence of Acute Kidney Injury in COVID-19

**DOI:** 10.3390/jcm9113547

**Published:** 2020-11-03

**Authors:** Ti-I Chueh, Cai-Mei Zheng, Yi-Chou Hou, Kuo-Cheng Lu

**Affiliations:** 1 Department of Medical Laboratory, Cardinal-Tien Hospital, New Taipei City 231, Taiwan; chiue0523@yahoo.com.tw; 2 Department of Education, Cardinal Tien Junior College of Healthcare and Management, New Taipei City 231, Taiwan; 3 Research Center of Urology and Kidney, Taipei Medical University, Taipei 110, Taiwan; 11044@s.tmu.edu.tw; 4 Division of Nephrology, Department of Internal Medicine, Taipei Medical University, Shuang Ho Hospital, Ministry of New Taipei City 235, Taiwan; 5 Division of Nephrology, Department of Internal Medicine, School of Medicine, College of Medicine, Taipei Medical University, Taipei 110, Taiwan; 6 Division of Nephrology, Department of Medicine, Cardinal-Tien Hospital, New Taipei City 231, Taiwan; athletics910@gmail.com; 7 School of Medicine, Fu Jen Catholic University, New Taipei City 242, Taiwan; 8 Division of Nephrology, Department of Medicine, Taipei Tzu Chi Hospital, Buddhist Tzu Chi Medical Foundation, New Taipei City 231, Taiwan

**Keywords:** COVID-19, acute kidney injury, ACE2, ADAM17, TMPRSS2, CD147

## Abstract

The coronavirus 2019 (COVID-19) pandemic has caused a huge impact on health and economic issues. Severe acute respiratory syndrome coronavirus 2 (SARS-CoV-2) causes cellular damage by entry mediated by the angiotensin-converting enzyme 2 of the host cells and its conjugation with spike proteins of SARS-CoV-2. Beyond airway infection and acute respiratory distress syndrome, acute kidney injury is common in SARS-CoV-2-associated infection, and acute kidney injury (AKI) is predictive to multiorgan dysfunction in SARS-CoV-2 infection. Beyond the cytokine storm and hemodynamic instability, SARS-CoV-2 might directly induce kidney injury and cause histopathologic characteristics, including acute tubular necrosis, podocytopathy and microangiopathy. The expression of apparatus mediating SARS-CoV-2 entry, including angiotensin-converting enzyme 2, transmembrane protease serine 2 (TMPRSS2) and a disintegrin and metalloprotease 17 (ADAM17), within the renal tubular cells is highly associated with acute kidney injury mediated by SARS-CoV-2. Both entry from the luminal and basolateral sides of the renal tubular cells are the possible routes for COVID-19, and the microthrombi associated with severe sepsis and the dysregulated renin–angiotensin–aldosterone system worsen further renal injury in SARS-CoV-2-associated AKI. In the podocytes of the glomerulus, injured podocyte expressed CD147, which mediated the entry of SARS-CoV-2 and worsen further foot process effacement, which would worsen proteinuria, and the chronic hazard induced by SARS-CoV-2-mediated kidney injury is still unknown. Therefore, the aim of the review is to summarize current evidence on SARS-CoV-2-associated AKI and the possible pathogenesis directly by SARS-CoV-2.

## 1. Introduction

The coronavirus disease 2019 (COVID-19) pandemic has had severe public health and economic impacts as a result of its rapid spread and association with severe acute respiratory distress syndrome. Severe acute respiratory syndrome coronavirus 2 (SARS-CoV-2) can lead to severe sepsis and systemic inflammation, which can induce multiple organ dysfunction. Acute kidney injury (AKI) is predictive of mortality and deteriorating organ dysfunction in patients with SARS-CoV-2 [1,2]. The most common risk factors related to SARS-Cov-2-mediated AKI include diabetes, obesity or hypertension and previous chronic kidney disease (CKD) [3]. Angiotensin-converting enzyme 2 (ACE2) and an assembly protein are known requirements for the entry of the virus into host cells. In patients with hypertension, diabetes mellitus and CKD, renin–angiotensin–aldosterone system activation is associated with ACE2 deficiency, which worsens renal tubulointerstitial fibrosis [4]. The modified phenotype of podocyte might facilitate SARS-CoV-2 entry into the reno-tubular system and worsen kidney damage. Moreover, SARS-CoV-2-associated sepsis might worsen AKI through the induction of a cytokine storm or hemodynamic dysregulation [5], but the pathogenesis and understanding of SARS-CoV-2-mediated kidney injury is still lacking. This review investigated the possible molecular mechanisms of acute kidney injury directly mediated by SARS-CoV-2.

## 2. High Levels of Angiotensin-Converting Enzyme 2 and Transmembrane Protease Serine 2 Expression in the Urinary System

According to data published by the Genotype-Tissue Expression project, ACE2 is highly expressed within the kidneys [6]. ACE2 is distributed throughout multiple cells within the kidneys and is most concentrated in proximal tubule cells [7]. Fan et al. suggested that specific ACE2 expression is high within proximal tubular cells but is not observed in immune cells or glomerular parietal epithelial cells [8]. Suryawanshi et al. analyzed the data of kidney tissues in single-cell RNA sequencing datasets and found that proximal tubular cells in the kidney co-express ACE2 and transmembrane protease serine 2 (TMPRSS2) [9]. Studies have reported high levels of ACE2 and TMPRSS2 expression in proximal renal tubular epithelial cells and kidney podocytes [10,11,12], and such characteristics facilitate the SARS-CoV replication within these cells [13].

## 3. TMPRSS2 and a Disintegrin and Metalloprotease 17 (ADAM17) Protect Against Viruses in Host Proteases

ACE2 within the cell membrane is important for SARS-CoV-2 entry into host cells [14]. However, in severe acute respiratory syndrome coronavirus 1 (SARS-CoV-1) or SARS-CoV-2 infection, poor prognosis is reportedly associated with ACE2 downregulation [15]. Theoretically, fewer viral entry routes should correspond to improved clinical outcomes. However, studies have demonstrated that decreased ACE2 expression levels lead to greater illness severity and more serious end-organ damage because ACE2 plays an anti-inflammatory role under renin–angiotensin–aldosterone system (RAAS) activation [16]. Classical RAAS involves the conversion of Angiotensin (Ang) I to Ang II and AngII binding with angiotensin-1 receptor (AT1R). The classic RAAS pathway involves vasoconstriction, oxidative stress, inflammation and fibrosis. The non-classical RAAS pathway, on the other hand, converts AngII to Ang(1–7) by ACE2, and the binding of Ang(1–7) with Mas receptor (MasR) provides vasodilatation and an anti-inflammatory effect [17]. Viral invasion into host cells occurs primarily through the ACE2 receptor; this receptor may mediate the entry of SARS-CoV-2 into host cells through two distinct routes. The first involves clathrin-dependent endocytosis and the second involves ACE2 receptor–mediated TMPRSS2-dependent membrane fusion [18].

**Figure 1:**
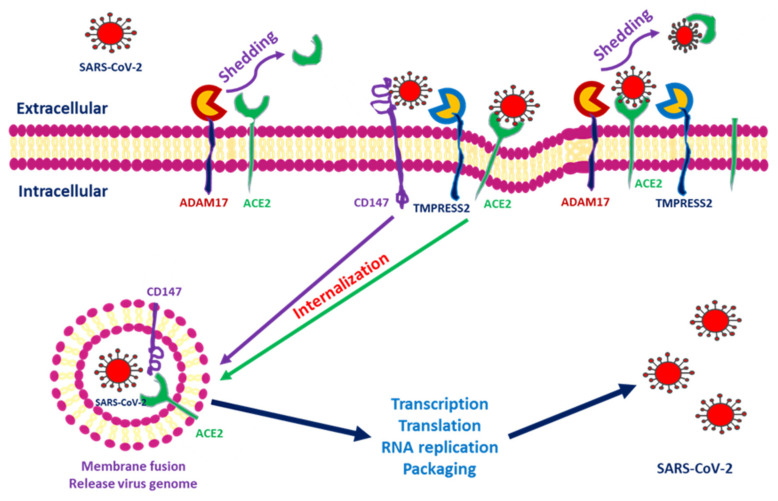
Severe acute respiratory syndrome coronavirus 2 (SARS-CoV-2) entry into cells through interactions among angiotensin-converting enzyme 2 (ACE2), transmembrane protease serine 2 (TMPRSS2), a disintegrin and metalloprotease 17 (ADAM17) and CD147. After SARS-CoV-2 interacts with the cells, ACE2 conjugates with the spike protein of SARS-CoV-2. TMPRSS2 interacts with the spike protein and facilitates internalization. ADAM17 serves to shed SARS-CoV-2 by transforming ACE2 into a soluble form. CD147 expressed within the transmembrane also facilitates entry and induces downstream inflammatory cytokine expression.

ACE2 downregulation occurs as these proteins are shed from cell membranes and circulated throughout the body. The end product of ACE2 cleavage mediated by a disintegrin and metalloprotease 17 (ADAM17) and TMPRSS2 may play a protective role against SARS-CoV-2 entry. In SARS-CoV-2, this occurs as a result of the spike protein activating ACE2 expression by seizing two host proteases: TMPRSS2, which facilitates viral entry by cleaving the S antigen into S1 (the active binding site), and ADAM17, which downregulates ACE2 expression by shedding ACE2 proteins into soluble form. The soluble ACE2 directly attached virus within circulation and decreased SARS-CoV-2 entry [19,20]. Research has indicated that ACE2 within human embryonic kidney cells can be shed by ADAM17 and protect against SARS-S entry [21]. In the human embryonic kidney cell line, decreased ACE2 expression is responsible for SARS-CoV-2 complications and end-organ damage and may cause greater harm to the host through enhanced ACE2 toxic effects, such as the activation of proinflammatory cytokines [22]. Heurich et al. suggested that TMPRSS2 and ADAM17 participate in the differential internalization of SARS-S [23]. TMPRSS2 cleaves ACE2 in arginine and lysine residues within amino acids 697 to 716, and the cleaved ACE2 is essential for SARS-S entry. Entry through TMPRSS2 is unrelated to ADAM17 because TRPMSS2 competes with ADAM17. Therefore, ADAM17 was proposed to participate in ACE2 ectodomain shedding, and TMPRSS2 enhances the intracellular cleavage of ACE2; thus, ADAM17 and TMPRSS2 compete during ACE2 processing. Hoffmann et al. demonstrated that TMPRSS2 inhibitors can block SARS-S cell entry through S protein processing [24]. Research has indicated that recombinant human ACE2 (rhACE2) mimics soluble ACE2 and inhibits viral invasion into host cells through competitive binding to CoV with the ACE2 lying in cellular membrane. The circulatory rhACE2-CoV2 binding decreases the internalization of membranous ACE2. Therefore, the non-classical RAAS provides further protection by alleviating intracellular inflammation [25]. Monteil et al. also used rhACE2 to block SARS-CoV-2 entry into the kidney organoid, demonstrating the possible protective effect of cleaved ACE2 against COVID-19 entry into host cells [26]. Therefore, in addition to its absolute expression, the cleavage and shedding of ACE2 could influence COVID-19 entry into cells (Figure 1).

However, more studies are necessary to clarify the role of ADAM17 and other proteases on ACE2 shedding in the kidney and the importance of these proteases in SARS-CoV-2 infection [27].

## 4. SARS-CoV-2 Invades Host Cells Through a Novel Route: CD147-Spike Protein

Research on kidney epithelial cells (Vero E6 cells) has demonstrated that spike proteins bound to CD147 can mediate SARS-CoV-2 host invasion. The localization of CD147 and spike protein was observed in SARS-CoV-2–infected cells through an immunoelectron microscope, and the discovery of the new CD147-SP route for SARS-CoV-2 invasion of host cells has provided a critical target for the development of a specific antiviral medicine [28].

### CD147 (Extracellular Matrix Metalloproteinase Inducer/Basigin) in Kidney Diseases

**Figure 2:**
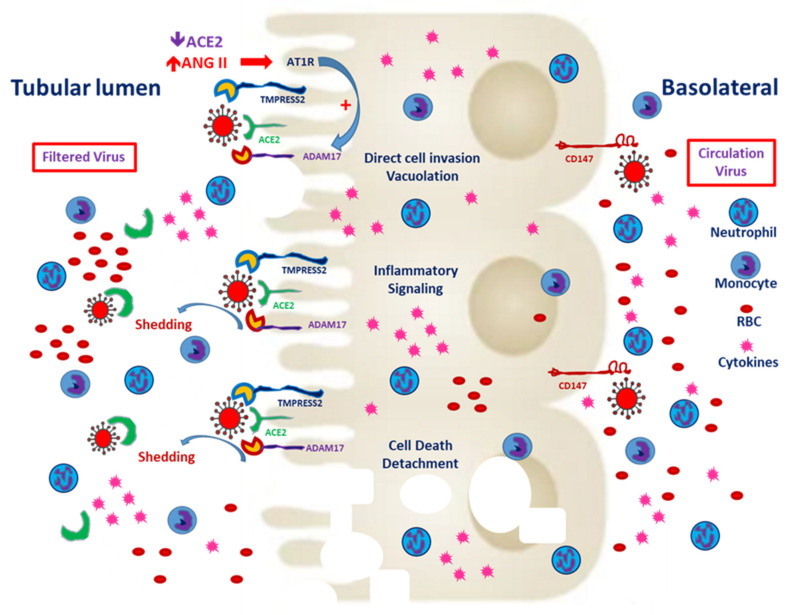
Severe acute respiratory syndrome coronavirus 2 (SARS-CoV-2) entry from the apical and basolateral sides of proximal tubular cells. CD147 expression is mainly distributed on the basolateral side of the proximal tubules. In the tubules predisposed to injury, enhanced CD147 might facilitate the entry of SARS-CoV-2. Classical RAAS activation is common in CKD patients, and local expression of angiotensin-converting enzyme 2 (ACE2) enhances hypercoagulability and induces microthrombi. Besides, filtered SARS-CoV-2 might enter the proximal tubules through ACE2 and TMPRESS2. Entry through either route could worsen tubular inflammation and increase cell death.

Basigin (Bsg)/CD147, also known as an extracellular matrix metalloproteinase (MMP) inducer (EMMPRIN), is the glycosylated transmembrane protein governing cell survival, cell migration and cancer invasion [29]. In SARS-CoV-2–mediated myocardial injury, EMMPRIN facilitates viral entry into the cardiomyocyte and induces the release of cytokines, such as interleukin-18 [30]. Moreover, the reactive oxygen species generated during inflammation dysregulate myocardial remodeling [31]. Atrial fibrillation is more inducible after SARS-CoV-2 entry and the downstream release of cytokines [32]. Wang et al. reported that the high affinity of CD147 and the spike protein to SARS-CoV-2 mediates viral entry into the kidney epithelium [28]. In normal kidneys, CD147 is highly expressed only on the basolateral side of tubular epithelial cells [29]. In the case of viremia, SARS-CoV-2 might invade renal proximal tubular epithelial cells through both the luminal surface and the basolateral side (Figure 2). Kato et al. demonstrated that Bsg/CD147 was associated with neutrophil recruitment in renal tubules with ischemic/reperfusion injury. In Bsg-deficient [Bsg(−/−)] mice, neutrophilic infiltration is suppressed after renal ischemic/reperfusion injury. Bsg/CD147 is a crucial physiologic ligand for E-selectin and therefore facilitates neutrophilic infiltration [33]. In an animal model of unilateral urinary obstruction, Bsg/CD147 promoted renal fibrosis by inducing MMP and hyaluronan expression. In a primary culture of the renal tubules of Bsg(−/−) mice, MMP expression was less responsive to transforming growth factor β [34].

The aforementioned research findings suggest that SARS-CoV-2 targets the renal tubules by entering from the basolateral side in adjunction with CD147. Yoshioka et al. reported that Bsg/CD147 is rarely expressed in healthy podocytes. In adriamycin-induced nephropathy, increases in Bsg expression and proteinuria are mediated by increases in focal adhesion kinase signaling [35]. In their clinical study, Musial et al. reported the serum concentrations of CD147 along with other fibrosis markers, such as transforming growth factor beta (TGF-β) levels, in children with CKD [36]. The findings indicated that patients with CKD may exhibit variations in CD147 expression that may contribute to pathological changes.

## 5. COVID-19 Prevents ACE2 from Converting ANG II into ANG I–VII and Increases Intracellular ANG II and Membrane ADAM17 Expression

Cytosolic ACE2 is a negative regulator for the activation of ACE and Ang I [37]. The carboxypeptidase ACE2 converts Ang II into Ang (1–7) and Ang I into Ang (1–9), thus preventing the conversion of Ang I into Ang II [38]. Under normal conditions, endothelial cells synthesize tissue factors and inhibitors of thrombosis to maintain immune and coagulation homeostasis [39]. COVID-19 enters cells through ACE2-mediated assembly. The inability of ACE2 to convert Ang I or Ang II when paired with COVID-19 results in endothelial senescence, caused by the upregulation of interleukin 6 and oxidative stress induced by disturbances in mitochondrial function [40]. In the hypoxia and sepsis, the un-countered AT1R activation by Ang II worsens the vasoconstriction and downstream fibrosis within lung tissue [37]. The excess Ang I and II induces immune and coagulation abnormalities and dysfunction. COVID-19/ACE2 complexes enter host cells through endocytosis, and those that are not endocytosed are shed by ADAM17 [40]. Increases in renal ADAM17 expression are known to mediate Ang II-induced growth of renal lesions in patients with CKD. The binding of Ang II to its AT1 receptor increases cytosolic ADAM17 expression and its translocation to the plasma membrane. In the vascular system, excessive ADAM17 expression induces the shedding of ACE2 and worsens further activation of the classical RAAS and inflammation [41]. ADAM17 induces the shedding of ACE2 and increases circulatory ACE2 (sACE2), thereby mitigating further entry of COVID-19 [42]. Additionally, ADAM17 modulates the shedding of transforming growth factor-α (TGF-α) from its transmembrane precursor and activates the epidermal growth factor receptor (EGFR), causing proteinuria, tubular hyperplasia, fibrosis and mononuclear cell infiltration [43]. TGF-α/EGFR-driven vitamin D receptor reduction impairs 1,25(OH)2D/VDR renoprotection [44]. ADAM17 is also expressed within the renal tubules, and its activation, mediated by classical RAAS activation, may prevent the COVID-19 shed by sACE2 from entering tubular cells [45]. However, this further activation of the classical RAAS may worsen the hypercoagulability of COVID-19 infection, thereby increasing the extent of tubular damage and the risk of damage to the interstitium.

## 6. Pathological Changes in AKI Mediated by COVID-19

As mentioned, COVID-19 can induce multiorgan dysfunction through direct transmission into cells via ACE2 or through the activation of a cytokine storm, which leads to the recruitment of inflammatory cells and activates apoptosis. AKI is common among patients with SARS-CoV-2, affecting up to 37%, and may occur concomitantly with the initiation of mechanical ventilation [46]. The mechanisms of AKI induced by SARS-CoV-2 also involve hemodynamic factors, direct cellular injury caused by SARS-CoV-2 invasion through ACE2 or the cytokine storm induced by SARS-CoV-2, because the SARS-CoV-2 could not be detected in the postmortem autopsy or in the graft of renal transplantation patients when using RNA in-situ hybridization or by immunochemistry for nucleocapsid [47,48,49]. Varga et al. suggested that SARS-CoV-2 is detectable within endothelial cells, reporting that in postmortem autopsy specimens, endotheliitis associated with inflammatory cell accumulation was common in different organs [50]. Pei et al. suggested that intrinsic AKI, rather than post- or pre-renal AKI, is the most common characteristic of renal involvement in patients with SARS-CoV-2 [51]. Therefore, understanding the role of AKI in COVID-19 is crucial for elucidating the pathology and clinical manifestations of COVID-19 (the summary is listed in the Table 1).

### 6.1. Acute Tubular Necrosis

Acute tubular necrosis is the most common pathological change observed in patients with COVID-19 who have AKI [52,53,54]. Hemodynamic instability, such as in patients with cardiorenal syndrome preceded by myocardial injury [5] or a cytokine storm induced by SARS-CoV-2 [55], reduces renal perfusion, and up to 80% of patients with acute tubular necrosis (ATN) require dialysis [52]. In patients with COVID-19 and ATN, proximal tubular cells might be the most susceptible tubular cells because of their high expression of ACE2 [53,56]. Werion et al. reported that tubular lumen dilatation with cellular debris, accompanied by changes in the brush border membranes of proximal tubules and moderate proteinuria, is a hallmark of COVID-19 involvement in the kidneys. In addition to increases in urinary β2-microglobulin or albumin, up to 46% of patients exhibit aminoaciduria [53]. Singer et al. demonstrated the interaction between amino acid transporter B^0^AT1 and ACE2 [57]. Altogether, these research findings highlight the importance of monitoring renal function in patients with COVID-19 recovering from AKI. In sum, the renal tubules, especially proximal tubules, carry CD147 within the basolateral side. The direct entry of SARS-CoV-2 through CD147 activates the partner protein of CD147, such as cyclophilins and integrins [54]. Cyclophilin then arouses the inflammation and worsens renal tubular damage [58]. Besides, the membrane attacking complex (MAC) has been detected within the renal tubules, which might be triggered by virus-mediated innate immunity [59,60,61].

### 6.2. Acute Interstitial Nephritis

Acute interstitial nephritis (AIN) is associated with the infiltration of inflammatory cells within the interstitium. The newly exposed antigen or hapten provokes inflammatory processes within tissues by recruiting cytotoxic T cells and antibody-producing B cells [62]. In patients with COVID-19, the infiltration of lymphocytes and macrophages within the tubulointerstitium were common when acute tubular necrosis developed [59]. According to the consensus report from Acute Disease Quality Initiative, the most common etiology of acute interstitial nephritis might be related to antibiotics or other nephrotoxic agents [3]. From the study by Diao et al., the CD68^+^ macrophages were the major inflammatory cells, whereas the role of CD4^+^ T cells or natural killer cells was less dominant [59]. Westhoff et al. reported the case of a patient receiving pancreas–kidney transplantation who developed AKI after COVID-19 infection; renal biopsy revealed mononuclear cell infiltration within the interstitium and in situ hybridization of SARS-CoV-2 [63]. Zhu et al. revealed the specific spectrum of immunity in patients with COVID-19. In single-cell sequencing of peripheral mononuclear cells, patients with COVID-19 had highly specific activation of STAT1 and IRF3 compared with patients with influenza A infection [64]. STAT-1 overexpression has been reported to be associated with tubulointerstitial fibrosis and epithelial to mesenchymal transition [65,66]. Moreover, one study demonstrated that the expression of CXCL-10+/CCL2+ macrophages induced by interferon gamma and tumor necrosis factor alpha might be related to tissue inflammation [67]. CD147 expression is also predictive of specific autoimmune-mediated nephritis. In sum, the CD147-mediated immune response in patients with COVID-19 is a possible pathogenesis of AIN [68].

### 6.3. Podocytopathy/Collapsing Focal Segmental Glomerulosclerosis 

**Figure 3:**
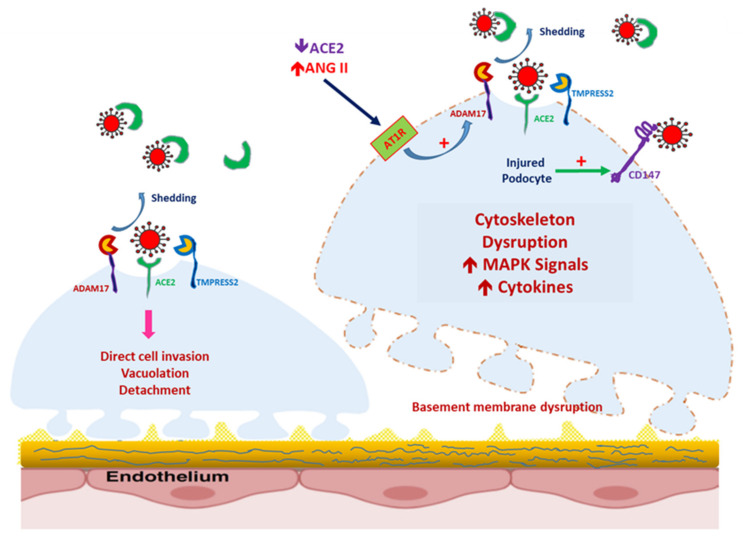
Severe acute respiratory syndrome coronavirus 2 (SARS-CoV-2)-induced podocyte injury, mediated by the increased expression of CD147. In healthy podocytes, CD147 is seldom expressed. In podocytes damaged by the overexpression of the renin–angiotensin–aldosterone system, CD147 expression is increased, even though a disintegrin and metalloprotease 17 (ADAM17) expression is also modulated by Ang II-mediated AT1R activation. SARS-CoV-2 can enter the podocyte and induce podocytopathy.

Along with the acute tubular necrosis with proximal tubular vacuolization, the podocyte could be a target for SARS-CoV-2 entry (Figure 3). Kissling et al. reported the case of an African male patient with AKI after SARS-CoV-2 infection; light microscopy revealed collapsing focal segmental sclerosis, and electron microscopy (EM) revealed viral inclusion within the vacuoles of the podocyte [69], in contrast to the EM result reported by Magoon et al. [70]. The spectrum of COVID-19-mediated podocytopathy remained unclear until the case report by Gupta et al. of a patient with repeat renal biopsy within 2 weeks. According to the report, an Asian male patient with an initial clinical presentation of nephrotic syndrome transitioned from minimal change disease to collapsing focal segmental glomerulosclerosis (FSGS) [71]. The mechanisms of collapsing FSGS and podocytopathy are still under investigation, but possibilities include cytokine storms and microRNA regulation [72]. High-risk groups for FSGS include patients who are APOL1 carriers [48]. Whether SARS-CoV-2-associated FSGS and human immunodeficiency virus-associated nephropathy (HIVAN) share the same pathomechanism is unknown. In patients with HIVAN, the loss of differentiating ability of podocyte gives the glomerulus a pseudo-crescent pattern, which involves collapsing FSGS with parietal epithelial cells [73,74]. ACE2 within the podocyte is known to play a protective role against specific renal diseases, such as diabetic nephropathy [75,76]; however, it remains unknown whether the polymorphism of ACE2 is predictive of SARS-CoV-2–associated FSGS or nephrotic syndrome. As mentioned, the expression of Bsg/CD147 is only increased in injured podocytes (Figure 3). In patients with a high risk of podocytopathy, such as those with diabetic nephropathy or obesity-related FSGS, the increased expression of CD147 in the podocyte might facilitate the entry of SARS-CoV-2 and increase injury [77].

### 6.4. Microangiopathy

COVID-19-associated coagulopathy has been widely reported to induce hypoxia, and downstream disseminated intravascular coagulation resulting from severe inflammation is a potential mechanism [78,79]. Thrombotic microangiopathy has been documented in patients with AKI and COVID-19. Jhaveri et al. noted microthrombi within the glomeruli in a patient with acute respiratory distress syndrome (ARDS) and AKI that was refractory to eculizumab treatment [80]. Mostly, the coagulation cascade is aroused by the SARS-CoV-2-mediated innate immunity, where MAC forms systemic thrombi/fibrin [60]. In the initial phase of the disease outbreak, a correlation was noted between SARS-CoV-2 and antiphospholipid antibodies. Zhang et al. noted the presence of anticardiolipin immunoglobulin A (IgA) antibodies and anti–β2-glycoprotein I IgA and IgG antibodies in patients with COVID-19 who had hypoxemic respiratory failure [81]. By contrast, Siguret et al. reported the presence of antiphospholipid antibodies in 12% of patients with COVID-19 [82]. Researchers have suggested that IgG in patients of COVID-19 could recruit neutrophilic aggregation with a similar ability to that of antiphospholipid antibody; moreover, a high incidence of thrombotic events in the inferior vena cava was noted in mice treated with the IgG of patients with COVID-19 [83]. In sum, activation of the classical RAAS and insufficient ACE2 may promote hypercoagulability [84,85]. It has been noticed that MAC was strongly detected in the tubules, but the involvement of MAC within the glomerulus or endothelium was less obvious [59,61]. However, MAC activity might triggered by the virus-mediated innate immunity within the endothelium [60]. Therefore, thrombotic microangiopathy within the kidney requires further investigation to provide a potential therapeutic target in COVID-19 patients with AKI.

## 7. ACEi/ARB Use and SARS-CoV-2-Associated AKI: Current Evidence

As explained in the previous section, ACE/Ang I directly activates vasoconstriction and downstream inflammation within the tissue. The application of ACEi/ARB could increase cytosolic ACE2 generation and lessen vasoconstriction and downstream tissue fibrosis [86]. In the scenario of acute kidney injury, the glomerular efferent arteriolar constriction mediated by Ang II is crucial for maintaining glomerular filtration during hemodynamic instability or vasodilatory shock [87,88]. From the report of Hirsch et al., chronic use of ACEi/ARB was a risk of acute kidney injury during SARS-CoV-2 infection [46]. The possible protective role of ACEi/ARB in lessening SARS-CoV-2 entry into lung tissue has been notified. Milne et al. demonstrated that in ACEi users, ACE2 and TMPRSS2 gene expression were lower than in non-ACEi users in the lung tissue [89], which was contrary with the increased ACE2 expression in the selected animal model [90,91]. Recently, Wysocki et al. provided in vivo evidence of the altered ACE2 expression in renal tubular cells after been treated with ACEi/ARB. The expression of ACE2 within apical membrane decreased in the ACEi/ARB-treated mice due to the internalization into cytosol [92]. Therefore, ACEi/ARB might not potentiate the hazard or disease severity in the SARS-CoV-2 infection. From the meta-analysis by Liu et al., patients with ACEi/ARB had lower disease severity and lower all-cause mortality [93]. Therefore, ACEi/ARB should only be discontinued if contraindications occur in the patients with SARS-CoV-2, such as hyperkalemia or unstable hemodynamics [94].

## 8. Conclusions

The COVID-19 pandemic has prompted the need for further understanding of how daily habits can influence multiple organ dysfunction by inducing severe inflammation or the direct invasion of SARS-CoV-2 into specific organs. Cell entry is mediated by ACE2 expression, the modulation of surface protein CD147 expression and the interplay between ADAM17 and TMPRSS2, which are commonly found in renal tubules. In SARS-CoV-2-associated AKI, virus-specific treatment might mitigate further cellular injury by influencing the protein associated with viral entry. Chronic follow-up for renal function in patients after SARS-CoV-2 infection should be important.

## Figures and Tables

**Table 1:** The histopathologic and clinical characteristics of acute kidney injury in COVID-19 patients and the possible pathogenesis based on current evidence.

Injured Site	Histologic Change	Postulated Pathogenesis	Clinical Manifestations	References
Acute tubular Necrosis	Tubular Lumen Dilatation with Cellular Debris Changes in The Brush Border Membranes of Proximal Tubules	Cytokine Storm and Hemodynamic Instability ACE2 Expression Within PT Enhances COVID-19 Entry CD147 Possibly Mediates Basolateral Entry Of COVID-19 Activation of Membrane Attack Complex Within Renal Tubules	Decrease In GFR Proteinuria	[5,28,55,59]
Acute Interstitial Nephritis	Mononuclear Cell Infiltration Within Interstitium	STAT1 And IRF3 Overexpression. Macrophage With CXCL-10+/CCL2+ Induce Cytokine Release	Decrease In GFR	[64,65,66,67]
Podocytopathy	Podocyte Foot Process Effacement Collapsing Glomerulus Pseudocrescent Formation	APOL1 Carriers Are Susceptible to Collapsing * Increased Expression of Bsg/CD147 in Injured Podocyte Enhances Further Viral Entry Damage Within Podocyte	Proteinuria	[73,74,77]
Thrombotic Microangiopathy	Microthrombi Within the Glomeruli	Anticardiolipin-Like Antibody Recruits Neutrophilic Aggregation Predisposing RAAS Activation Decreases ACE2 And Enhances Hypercoagulopathy Membrane Attack Complex Activation Within Endothelium	Decrease In GFR Hematuria	[60,80,81,83]

* Collapsing glomerulopathy is characterized by segmental or global glomerular tuft collapse with hypertrophy and hyperplasia of the overlying podocytes and dedifferentiated podocytes or parietal epithelial cells. Along with acute tubular injury, tubular dilation with microcyst formation and interstitial inflammation are common.

## Abbreviations

ACE2	Angiotensin-Converting Enzyme 2
ADAM17	A Disintegrin and Metalloprotease 17
AKI	Acute Kidney Injury
Ang	Angiotensin
APOL1	Apolipoprotein L1
ARDS	Acute Respiratory Distress Syndrome.
AT1R	Angiotensin Type 1 Receptor
COVID-19	Coronavirus 2019
C/EBPβ	CAAT-Enhancer Binding Protein-B
FSGS	Focal Segmental Glomerular Sclerosis
IRF3	IFN Regulatory Factor 3
MasR	Mas Receptor
MMP	Matrix Metalloproteinase
NEP	Neprilysin
SARS-CoV-2	Severe Acute Respiratory Syndrome Coronavirus 2
SHPT	Secondary Hyperparathyroidism
STAT1	Signal Transducer and Activator of Transcription 1
TGF-α	Transforming Growth Factor-A
TMPRSS2	Transmembrane Protease Serine 2
